# Phase III AIRTIVITY study design: verducatib (BI 1291583) in people with bronchiectasis

**DOI:** 10.1183/23120541.01068-2025

**Published:** 2026-09-01

**Authors:** James D. Chalmers, Michal Shteinberg, Sanjay H. Chotirmall, Wiebke Sauter, Patrick A. Flume, Raksha Jain, Kozo Morimoto, Anne E. O'Donnell, Felix C. Ringshausen, Henrik Watz, Jin-Fu Xu, Johanna Rauch, Anastasia Eleftheraki, Annemarie T. Engel, Anne B. Chang, Kim G. Nielsen, Giselle Benitez, Abhya Gupta, Marcus A. Mall, Pamela J. McShane

**Affiliations:** 1 Radcliffe Department of Medicine, University of Oxford, Oxford, UK; 2 Pulmonology Institute and Cystic Fibrosis Center, Carmel Medical Center, Haifa, Israel; 3 Technion – Israel Institute of Technology, The B. Rappaport Faculty of Medicine, Haifa, Israel; 4 Lee Kong Chian School of Medicine, Nanyang Technological University, Singapore; 5 Department of Respiratory and Critical Care Medicine, Tan Tock Seng Hospital, Singapore; 6 Boehringer Ingelheim International, Ingelheim am Rhein, Germany; 7 Medical University of South Carolina, Charleston, SC, USA; 8 Department of Internal Medicine, University of Texas Southwestern Medical Center, Dallas, TX, USA; 9 Respiratory Disease Center, Fukujuji Hospital, Japan Anti-Tuberculosis Association, Tokyo, Japan; 10 Georgetown University Medical Center, Washington, DC, USA; 11 Department of Respiratory Medicine and Infectious Diseases, Hannover Medical School (MHH), Hannover, Germany; 12 Biomedical Research in End-Stage and Obstructive Lung Disease Hannover (BREATH), German Center for Lung Research (DZL), Hannover, Germany; 13 European Reference Network on Rare and Complex Respiratory Diseases (ERN-LUNG), Frankfurt, Germany; 14 Velocity Clinical Research Germany GmbH, Ahrensburg, Germany; 15 Department of Respiratory Medicine, University Hospital Schleswig-Holstein, Campus Lübeck, Airway Research Center North (ARCN), German Center for Lung Research (DZL), Lübeck, Germany; 16 Department of Respiratory and Critical Care Medicine, Tongji Hospital, Tongji University, Shanghai, China; 17 Boehringer Ingelheim Pharmaceuticals, Ridgefield, CT, USA; 18 Boehringer Ingelheim Pharma, Ingelheim am Rhein, Germany; 19 Australian Centre for Health Services Innovation and School of Medicine, Queensland University of Technology, Brisbane, Queensland, Australia; 20 Queensland Children's Hospital, Brisbane, Queensland, Australia; 21 Danish PCD Centre, Paediatric Pulmonary Service, Department of Paediatrics and Adolescent Medicine, Copenhagen University Hospital, Rigshospitalet, Copenhagen, Denmark; 22 The Primary Ciliary Dyskinesia Clinical Trial Network (PCDCTN), Copenhagen University Hospital, Rigshospitalet, Copenhagen, Denmark; 23 Department of Clinical Medicine, University of Copenhagen, Copenhagen, Denmark; 24 Boehringer Ingelheim Pharma, Biberach an der Riss, Germany; 25 Department of Pediatric Respiratory Medicine, Immunology and Critical Care Medicine, Charité – Universitätsmedizin Berlin, Berlin, Germany; 26 German Center for Lung Research (DZL), associated partner site Berlin; 27 German Center for Child and Adolescent Health (DZKJ), partner site Berlin, Berlin, Germany; 28 Cluster of Excellence ImmunoPreCept, Charité – Universitätsmedizin Berlin, Berlin, Germany; 29 Critical Care Medicine and Pulmonary Branch, National Heart, Lung, and Blood Institute, National Institutes of Health, Bethesda, MD, USA

## Abstract

There is an unmet need for therapies targeting chronic neutrophilic inflammation in bronchiectasis. In the phase II, dose-finding AIRLEAF study, the dipeptidyl peptidase 1 (cathepsin C) inhibitor verducatib (BI 1291583) reduced the pulmonary exacerbation risk in people with bronchiectasis and demonstrated a similar safety profile to placebo; these results support the investigation of verducatib in a phase III study. Here, we describe the design of the randomised, placebo-controlled phase III AIRTIVITY study (ClinicalTrials.gov identifier NCT06872892), assessing the efficacy, safety and tolerability of verducatib in people with bronchiectasis.

In AIRTIVITY, approximately 1680 adults and 75 adolescents (aged 12–<18 years) with bronchiectasis with a broad range of aetiologies are randomised 2:1 to receive oral verducatib 2.5 mg or placebo for at least 52 weeks and up to 76 weeks. Unlike previous studies of bronchiectasis, people with cystic fibrosis bronchiectasis are included. The primary objective is to demonstrate the superiority of verducatib compared with placebo on the annualised rate of pulmonary exacerbations up to 76 weeks. The main secondary objective is to demonstrate a reduction in lung function decline, an improvement in respiratory symptoms, a prolongation in time to first pulmonary exacerbation and a reduction in annualised rate of severe pulmonary exacerbations for verducatib *versus* placebo. All pulmonary exacerbations are adjudicated.

If the efficacy of verducatib is demonstrated and there is a favourable benefit–risk ratio, the AIRTIVITY results will provide confirmatory evidence for the use of verducatib 2.5 mg in a large and broad bronchiectasis population, independent of underlying aetiology.

## Shareable abstract

**AIRTIVITY is a randomised phase III study assessing the efficacy, safety and tolerability of verducatib (BI 1291583; dipeptidyl peptidase 1 inhibitor) 2.5 mg *versus* placebo in people with bronchiectasis of various aetiologies, including cystic fibrosis** https://bit.ly/3Qm76ld

## Graphical abstract

**Figure:**
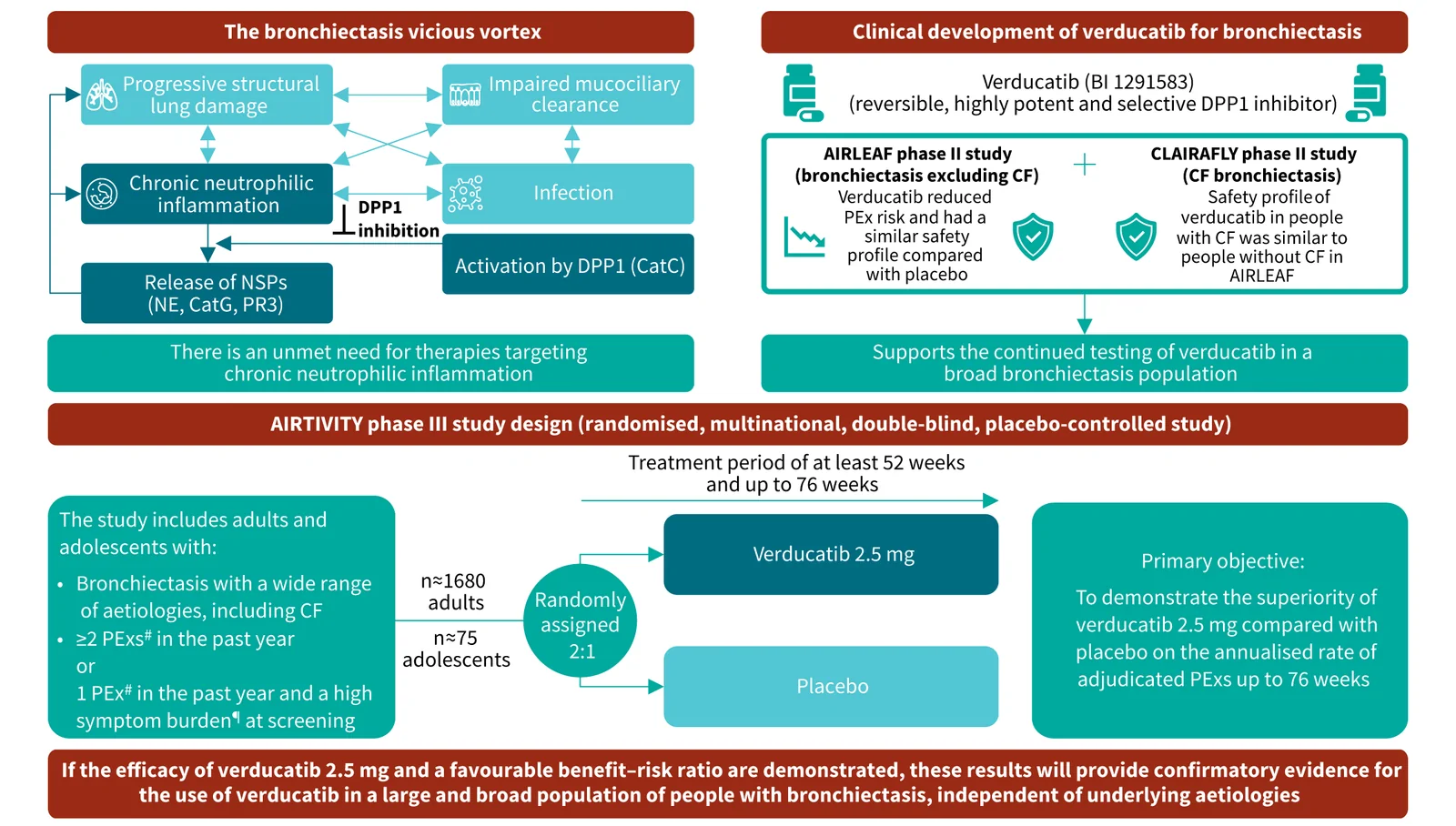
Overview of the key elements of the AIRTIVITY study design. CatC: cathepsin C; CatG: cathepsin G; CF: cystic fibrosis; DPP1: dipeptidyl peptidase 1; NE: neutrophil elastase; NSP: neutrophil serine protease; PEx: pulmonary exacerbation; PR3: proteinase 3; SGRQ: St George's Respiratory Questionnaire. ^#^: Requiring antibiotic treatment. ^¶^: SGRQ Symptoms score >40 for adults or according to the investigator for adolescents.

## Introduction

Bronchiectasis, a chronic inflammatory lung disease, is characterised by abnormal and permanent dilatation of the airways, sputum production and chronic cough [1, 2]. Frequent pulmonary exacerbations (PExs), common in people with bronchiectasis, are associated with poor quality of life, daily symptoms, hospitalisation and mortality [2]. Bronchiectasis is the third most common chronic airway disease globally, and its prevalence is increasing [3–5]. It is more common in females and older patients [5, 6] and imposes a considerable burden on patients and healthcare systems [7, 8]. Although bronchiectasis is most prevalent in adults, it also occurs in children and adolescents [9]. Despite the increasing recognition of the disease in adolescents [10, 11], it is frequently underdiagnosed or diagnosed late in this younger population, often only being recognised once they reach adulthood [12]. Bronchiectasis presents heterogeneously given its association with various aetiologies and close links with a variety of diseases, including, but not exclusive to, post-infection (including pneumonia, tuberculosis and non-tuberculous mycobacterial infection), asthma, COPD, immunodeficiency, autoimmune conditions (*e.g.* rheumatoid arthritis) and genetic conditions (*e.g.* primary ciliary dyskinesia and cystic fibrosis; CF) [8, 13].

Chronic neutrophilic airway inflammation is an integral feature of bronchiectasis regardless of aetiology [1, 14, 15]. The activation of neutrophils in the airways releases high amounts of neutrophil serine proteases (NSPs), such as neutrophil elastase (NE), proteinase 3 (PR3) and cathepsin G [16]. Excessive NSP activity can cause an imbalance between NSPs and their antiproteases, which is fundamental in the pathogenesis of bronchiectasis and contributes to structural lung damage, mucus hypersecretion, impaired mucociliary clearance and chronic infection [13, 15]; these factors create a vicious vortex, with concurrent rather than sequential roles in disease pathogenesis, resulting in disease progression and PExs (figure 1) [13, 15, 17, 18].

**FIGURE 1:**
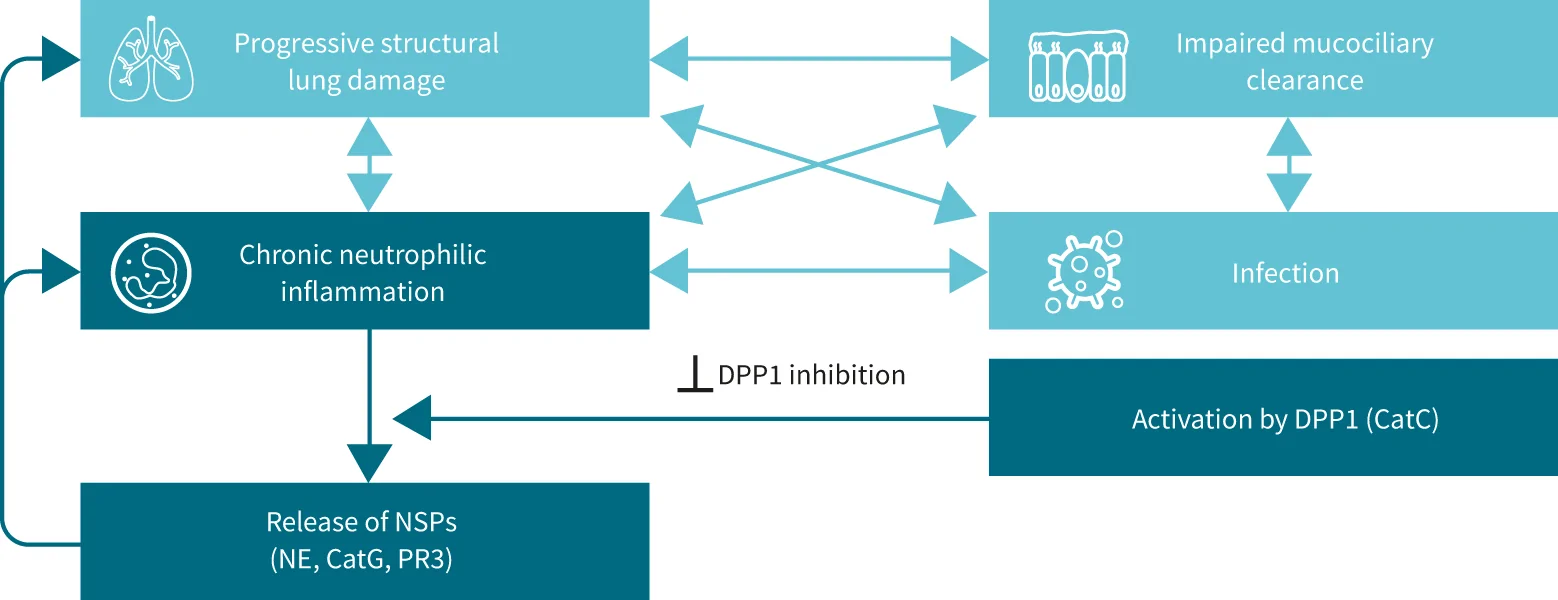
The bronchiectasis vicious vortex. CatC: cathepsin C; CatG: cathepsin G; DPP1: dipeptidyl peptidase 1; NE: neutrophil elastase; NSP: neutrophil serine protease; PR3: proteinase 3.

Bronchiectasis is an inflammatory rather than purely infectious disease, with most therapies to date focusing on symptom control and treating exacerbations [1, 18]. In people with CF bronchiectasis (CFBE), the development of cystic fibrosis transmembrane conductance regulator modulator therapy (CFTRmt) has improved lung function and reduced PEx rate, but not all people with CFBE are eligible for CFTRmt [19–21]. Furthermore, it has been shown that airway inflammation in people with CF receiving CFTRmt can persist at levels seen in people with bronchiectasis caused by other aetiologies [20, 21], and baseline NE activity has been shown to be comparable between people with bronchiectasis caused by CF and other causes [22, 23]. Therefore, an unmet need exists for therapies that target the chronic neutrophilic inflammation underpinning bronchiectasis across all aetiologies.

The cysteine protease dipeptidyl peptidase 1 (DPP1; or cathepsin C) is responsible for activating NSPs during neutrophil maturation in the bone marrow [24, 25]. DPP1 inhibition is a treatment strategy to reduce NSP activity in people with bronchiectasis. Verducatib (BI 1291583) is a reversible, highly selective and potent inhibitor of DPP1 [26]. In pre-clinical studies, verducatib was shown to have a high bone-marrow-to-plasma distribution ratio and dose-dependently reduced NE and PR3 activity [26]. Phase I studies demonstrated the safety and tolerability of verducatib in healthy volunteers across tested doses and that it is readily absorbed, with no serious adverse events (AEs) or deaths reported [27]. Additionally, verducatib dose-dependently inhibited DPP1, reduced downstream NE activity and decreased PR3 levels. The phase II, dose-finding AIRLEAF study (NCT05238675) demonstrated that verducatib reduced PEx risk in adults with bronchiectasis caused by aetiologies other than CF. The 2.5 mg dose of verducatib had similar or improved outcomes across different end-points *versus* the 5 mg dose and a comparable safety profile to placebo [22]. Treatment benefit was similar between people with multiple PExs (≥2) in the last 12 months and people with 1 PEx in the last 12 months who had a high symptom burden (St George's Respiratory Questionnaire (SGRQ) Symptoms score >40). CLAIRAFLY (phase II; NCT05865886) demonstrated that inhibition of sputum NE activity by verducatib (5 mg) in people with CFBE was comparable to that seen in people with bronchiectasis caused by other aetiologies in AIRLEAF, with a similar safety profile [22, 23]. The CLAIRLEAF study (NCT05846230) is ongoing, investigating longer-term treatment with verducatib in people with bronchiectasis with or without CF who had completed either phase II study. Findings from these phase II studies support the continued clinical development of verducatib 2.5 mg in a broad bronchiectasis population.

Here, we present the design of the phase III AIRTIVITY study (NCT06872892), assessing the efficacy, safety and tolerability of verducatib 2.5 mg in people aged ≥12 years with bronchiectasis with a wide range of aetiologies. A unique attribute of AIRTIVITY, compared with previous studies of bronchiectasis, is that people with CFBE are also included. In addition to people with ≥2 PExs requiring antibiotic treatment in the last 12 months, people with 1 PEx requiring antibiotic treatment and a high symptom burden are eligible for enrolment. The aetiology-agnostic approach, and the inclusion of people with just one PEx and a high symptom burden, are two key differentiators of the AIRTIVITY study design compared with previous studies of bronchiectasis [22, 28–30]. The study started in June 2025 and will be conducted at approximately 500 sites (academic and community centres) across approximately 50 countries.

## Methods

### Study design

The protocol is structured according to the Standard Protocol Items: Recommendations for Interventional Trials (SPIRIT 2013 Checklist) [31].

AIRTIVITY is a randomised, prospective, multinational, multicentre, double-blind, placebo-controlled phase III study, assessing approximately 1680 adults (∼240 with CF) and 75 adolescents (aged 12–<18 years and weighing ≥35 kg; up to 30% with CF) with bronchiectasis. Participants are randomised 2:1 to receive oral verducatib 2.5 mg or placebo once daily. For adults, randomisation is stratified by *Pseudomonas* *aeruginosa* status at screening (positive/negative), number of PExs in the year prior to screening (1/2/≥3), geographic region (Eastern Europe/Asia/rest of the world) and CF status (CF/no CF). Adults with CF are further stratified by receipt of standard-of-care CFTRmt (yes/no). For adolescents, randomisation is only stratified by CF status (CF/no CF). The study comprises a screening period lasting up to 6 weeks, with the possibility to extend up to an additional 4 weeks under specific circumstances (figure 2).

**FIGURE 2:**
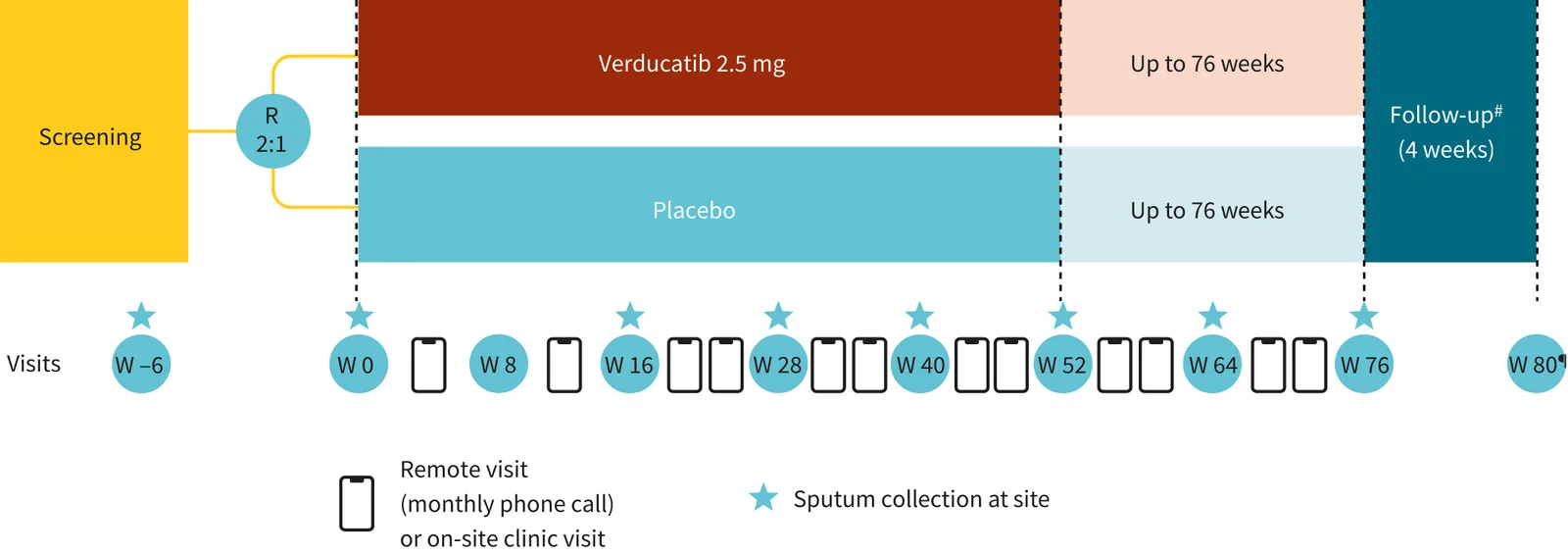
AIRTIVITY study design. ^#^: Participants may have the option to transfer to an open-label extension study. ^¶^: W 80 visit for all adolescents and for adults not participating in the open-label extension study and/or who have a drug-related adverse event at the end of treatment. R: randomisation; W: week.

For all participants, the treatment period lasts for at least 52 weeks and up to a maximum of 76 weeks, depending on time of enrolment. In-person visits occur at screening, randomisation, end of treatment and at weeks 8, 16 and every 12 weeks thereafter until week 76. Between these site visits, participants can choose to have remote (monthly phone calls) or further onsite clinic visits. The study will conclude when the last-enrolled participant reaches week 52. Participants who discontinue study treatment prematurely are encouraged to attend all remaining planned visits. Participants who complete treatment as planned may have the option to roll over into an open-label extension study.

### Key eligibility criteria

Prior to study enrolment, participants provide written informed consent or assent, if applicable, in accordance with good clinical practice and local legislation. At screening, participants must be aged ≥12 years (adolescents must weigh ≥35 kg at visit 1), have computed tomography-confirmed bronchiectasis, and have a history of PExs requiring antibiotic treatment in the year before visit 1 (either two or more PExs, one PEx plus SGRQ Symptoms score >40 (adults only) or one PEx plus a high symptom burden according to the investigator (adolescents only)) (table 1). For those receiving stable oral or inhaled antibiotics as a long-term bronchiectasis treatment and those receiving CFTRmt as a CF treatment, ≥1 PEx must have occurred since initiation of these treatments. Adults should be able to produce sputum during screening. For adolescents, sputum is requested but not required.

**TABLE 1:** All eligibility criteria

Inclusion criteria	Exclusion criteria
Male or female (WOCBP^#^ must be ready and able to use highly effective methods of birth control (per ICH M3 (R2)) that result in a low failure rate of <1% per year when used consistently and correctly, as well as one barrier method) Signed and dated written informed consent and assent, if applicable, prior to admission to the study, in accordance with GCP and local legislation Aged ≥12 years when signing informed consent/assent • Those aged 12–<18 years must weigh ≥35 kg at visit 1 Clinical history consistent with bronchiectasis (*e.g.* cough, chronic sputum production and recurrent respiratory infections) and investigator-confirmed diagnosis of bronchiectasis by CT scan^¶^ Adult participants should be able to produce sputum for screening History of documented PExs requiring antibiotic treatment; in the 12 months before visit 1, participants must have had ≥2 PExs (all participants), 1 PEx and a St George's Respiratory Questionnaire Symptoms score >40 at screening visit 1 (participants aged ≥18 years only) or 1 PEx and a high symptom burden according to the investigator (participants aged 12–<18 years only)^+^	Evidence or medical history of moderate or severe liver disease (Child–Pugh score B or C hepatic impairment) Absolute blood neutrophil count of <1000·mm^−3^ (equivalent to <1000 cells·μL^−1^ or <10^9^ cells·L^−1^) at visit 1 eGFR according to CKD-EPI formula (adults) or CKiD-U25 (adolescents) <30 mL·min^−1^ at visit 1 Total IgG levels <5 g·L^−1^ at visit 1 Any findings in the medical examination and/or laboratory value assessed at screening visit 1 or during screening period that, in the opinion of the investigator, may put the participant at risk by participating in the study Relevant chronic or acute infections including, but not limited to, HIV and viral hepatitis^§^ Any new or newly diagnosed condition of primary or secondary immunodeficiency within 1 year of randomisation a) People with primary or secondary immunodeficiency (*e.g.* CVID or hypogammaglobulinaemia) diagnosed >1 year prior to randomisation can be included if their condition is considered stable (per the ISF) for the last year prior to randomisation (*i.e.* people must not have had severe or life-threatening infection events and are stabilised by adequate maintenance treatment or do not require treatment in the year prior to randomisation as per current guidelines) b) Eligible participants with immunodeficiencies must maintain standard-of-care monitoring and treatment by a specialist who is informed about the study participation, and receive standard-of-care treatment for intercurrent infections, must be fully vaccinated according to local guidelines prior to visit 1 (*e.g.* CDC immunisation schedule) and must be willing to maintain adequate vaccination status for the duration of the study Allergic bronchopulmonary aspergillosis that is being treated or requires treatment Palmoplantar keratoderma, keratoderma climactericum or hyperkeratosis, unless clinically stable for ≥3 months before randomisation Tuberculosis or non-tuberculosis mycobacterial infection which is being treated or requires treatment according to local guidelines (laboratory tests (*e.g.* Quantiferon Gold test) may be performed at the discretion of the investigator) Any clinically relevant (at the discretion of the investigator) acute respiratory infection or ongoing PEx at screening visit 1 or during the screening period unless recovered prior to visit 2 (in the opinion of the investigator) Any relevant pulmonary, gastrointestinal, hepatic, renal, cardiovascular, metabolic, immunological, hormonal or other disorder that may put the participant at risk by participating in the study (in the opinion of the investigator) Any evidence of Papillon–Lefèvre syndrome Participants who must or wish to continue the intake of restricted medications (defined in the protocol) or any drug considered likely to interfere with the safe conduct of the study Major surgery (major according to the investigator's assessment) performed in the 6 weeks prior to randomisation or scheduled during study period Any documented active or suspected malignancy or history of malignancy within 5 years prior to screening, except appropriately treated *in situ* non-melanoma skin cancers or *in situ* carcinoma of the uterine cervix Participants not expected to comply with the protocol requirements or not expected to complete the study as scheduled, *i.e.* chronic alcohol or drug abuse or any other condition that, in the investigator's opinion, makes the participant an unreliable study participant Significant haemoptysis (≥300 mL and/or requiring blood transfusion) in the 4 weeks prior to screening visit 1 (and during screening phase) Current tobacco smoker, stopped smoking within 3 months of screening, or not willing to maintain nonsmoking status for the duration of the study Currently enrolled in another study of an investigational device or drug, or <30 days or <5 half-lives, whichever is longer, prior to visit 2 since ending another study of an investigational device or drug or receiving other investigational treatment(s) Previous treatment with a DPP1 inhibitor^ƒ^ Women who are pregnant, nursing or who plan to become pregnant while in the study Any contraindication to the class of drug under study, including known hypersensitivity to the drug or its excipients Participants who have received a transplant or are likely to require transplantation during the study period as determined by the investigator

CDC: Centers for Disease Control and Prevention; CFTRmt: cystic fibrosis transmembrane conductance regulator modulatory therapy; CKD-EPI: Chronic Kidney Disease-Exocrine Pancreatic Insufficiency; CT: computed tomography; CVID: common variable immunodeficiency; DPP1: dipeptidyl peptidase 1; eGFR: estimated glomerular filtration rate; GCP: good clinical practice; HIV: human immunodeficiency virus; ICH: International Council for Harmonisation of Technical Requirements for Pharmaceuticals for Human Use; IgG: immunoglobulin G; ISF: investigator site file; PEx: pulmonary exacerbation; WOCBP: women of childbearing potential. ^#^: Women are considered WOCBP following menarche and until becoming post-menopausal unless permanently sterile. Permanent sterilisation methods include hysterectomy, bilateral salpingectomy and bilateral oophorectomy. Tubal ligation is not considered to be a method of permanent sterilisation. ^¶^: Participants whose past CT scan image records are not available will undergo a chest CT scan during screening. Historical scans must not be older than 5 years. ^+^: For participants receiving stable oral or inhaled antibiotics as chronic treatment for bronchiectasis and participants receiving CFTRmt for cystic fibrosis, ≥1 PEx must have occurred since initiation of stable antibiotics or CFTRmt. ^§^: Corresponding laboratory tests will be performed during screening. ^ƒ^: Participants who were randomised into prior studies with a DPP1 inhibitor and only received placebo are eligible for this study.

Enrolment is permitted for people with non-tuberculosis mycobacterial infection or allergic bronchopulmonary aspergillosis that is not being treated or requiring treatment, and those with clinically stable (for ≥3 months before randomisation) palmoplantar keratoderma. Those with any clinically relevant acute respiratory infection during screening are eligible for inclusion if they have recovered, in the opinion of the investigator, prior to visit 2; only following recovery can the participant be randomised. Similarly, people with primary or secondary immunodeficiency, if clinically stable for the last year prior to randomisation, are eligible, but those diagnosed within 1 year of randomisation are ineligible. Those on immunoglobulin replacement therapy with a stable treatment schedule for the last year, and people on stable treatment with immunosuppressants for the last year, are also eligible, unless meeting other exclusion criteria. People with Papillon–Lefèvre syndrome (PLS) are also ineligible. Notably, people with CF, asthma or COPD, as well as people receiving stable concomitant immunosuppressants for >1 year or who stopped receiving immunosuppressants >1 year prior to visit 2, are eligible for enrolment.

To best reflect the treatment benefit of verducatib on top of standard-of-care treatments, concomitant long-term oral or inhaled antibiotic or CFTRmt treatment is permitted if these treatments began before the study and have been stable for >3 months or >12 months, respectively. Additionally, treatments such as oral or parenteral corticosteroids can be used with restrictions, if given prior to the first dose or concomitantly. Bronchodilators are permitted.

### Objectives and end-points

The primary objective is to demonstrate superiority of verducatib 2.5 mg compared with placebo on the primary end-point, the annualised rate of adjudicated PExs over a treatment period of up to 76 weeks.

The main secondary objective is to demonstrate the following for verducatib 2.5 mg compared with placebo: a reduction in lung function decline, as measured by absolute change from baseline in post-bronchodilator per cent predicted forced expiratory volume in 1 s and forced vital capacity at week 52; an improvement in respiratory symptoms, as measured by absolute change from baseline in Quality of Life Questionnaire-Bronchiectasis (QOL-B) respiratory symptoms domain score at week 52; a reduction in the risk of an adjudicated PEx, as measured by time to first adjudicated PEx up to week 76; and a reduction in the annualised rate of severe adjudicated PExs up to week 76.

Exploratory end-points include pharmacokinetic and biomarker assessments, and further clinical outcome assessments. Table 2 presents all primary and main secondary end-points.

**TABLE 2:** Primary and main secondary end-points

**Primary end-point**	Annualised rate of adjudicated PExs (number of events per person year) up to week 76
**Main secondary end-points**	Absolute change from baseline in post-bronchodilator FEV_1_ (% predicted) at week 52 Absolute change from baseline in post-bronchodilator FVC (% predicted) at week 52 Absolute change from baseline in QOL-B respiratory symptoms domain score at week 52 Time to first adjudicated PEx up to week 76 Annualised rate of severe adjudicated PExs up to week 76

FEV_1_: forced expiratory volume in 1 s; FVC: forced vital capacity; PEx: pulmonary exacerbation; QOL-B: Quality of Life Questionnaire-Bronchiectasis.

### Assessments

A PEx is defined per the European Multicentre Bronchiectasis Research Collaboration and the US Bronchiectasis and NTM Association consensus criteria [32] as having ≥3 of the following symptoms for ≥48 h, resulting in an antibiotic prescription: increased cough, increased sputum volume or change in sputum consistency, increased sputum purulence, increased breathlessness and/or decreased exercise tolerance, fatigue and/or malaise, or haemoptysis for all study participants. All PExs are adjudicated. A severe PEx is defined as a PEx that results in hospitalisation and/or intravenous antibiotic administration, or with a fatal outcome. For adolescents, PEx events are also collected as per the European Respiratory Society paediatric bronchiectasis definition for use in clinical studies for further analyses [10]. Participants fill in a daily diary (Bronchiectasis Exacerbation and Symptom Tool) at home, helping investigators identify PEx events and changes in symptoms [33]. Pulmonary function is assessed by spirometry. Safety is monitored throughout the study by clinical assessment, AE reporting and monitoring of safety laboratory parameters; for adolescents, changes in growth and pubertal development are also monitored. Quality of life is assessed using patient-reported outcome measures at specific onsite visits, including QOL-B respiratory symptoms domain, Chronic Airways Assessment Test, EuroQol Five-Dimensional Five-Level Quality of Life Questionnaire (both adults only), EuroQol Five-Dimensional Youth Three-Level QoL Questionnaire and Paediatric QoL Inventory (both adolescents only). Biomarkers are analysed in the sputum (*e.g.* NE activity), plasma, serum and urine (adolescents only). Pharmacokinetics are assessed using blood samples.

### Sample size and statistical analysis

This study has been designed to have approximately 90% power to detect a 25% reduction in the annualised rate of adjudicated PExs, up to 76 weeks at a two-sided α of 0.125%. The rate of adjudicated PExs is assumed to follow a negative binomial model. The sample size of 75 adolescents is adequate to evaluate the safety of verducatib in this population and to observe directional trends in efficacy. Pre-specified subgroup analyses of the primary end-point are conducted in various subgroups defined by key demographic and disease characteristics, including sex, age, geographical region, number of PExs in the year prior to screening, aetiology of bronchiectasis (CF *versus* not CF) and *P.* *aeruginosa* status at baseline.

## Discussion

Bronchiectasis is an inflammatory disease rather than a purely infectious disease; hence, therapies targeting neutrophilic inflammation, a common feature of symptomatic disease, are needed. Development of verducatib aims to address this unmet need. Given the promising results from pre-clinical, phase I and phase II studies of verducatib [22, 23, 26, 27], AIRTIVITY intends to confirm the efficacy, safety and tolerability of verducatib 2.5 mg in a large and broad bronchiectasis population aged ≥12 years, irrespective of underlying aetiologies. Study results may also support the concept of treating people with bronchiectasis with a high symptom burden, irrespective of PEx frequency. Additionally, the inclusion of adolescents will generate data that could inform the future development of verducatib, including the selection of dose, for all adolescents with bronchiectasis. AIRTIVITY is the first interventional study to investigate such a wide range of bronchiectasis aetiologies, including CF, reflecting the real-world bronchiectasis population.

The dose of verducatib 2.5 mg was selected following AIRLEAF (phase II; dose-finding), as 2.5 mg showed similar or improved efficacy outcomes across different end-points *versus* 5 mg and a similar safety profile to placebo [22]. Findings from recent phase II studies have provided the rationale for inclusion of a broad population of participants with bronchiectasis in AIRTIVITY: AIRLEAF supported the inclusion of people with bronchiectasis of various aetiologies, including those traditionally not included in such trials, such as people with a primary diagnosis of asthma or COPD [22, 28, 29]; CLAIRAFLY supported the inclusion of those with CF [23]. A single definition for PExs is used for all participants, irrespective of aetiology, to ensure consistency; the European Multicentre Bronchiectasis Research Collaboration and the US Bronchiectasis and NTM Association consensus criteria, which have been used widely across bronchiectasis studies, are expected to encompass events meeting the Fuchs criteria, which are typically used in studies of CF. Similarly, the patient-reported outcome measures used in the study have been deemed appropriate for use in people with CFBE, as well as in those with bronchiectasis caused by other aetiologies [34]. The sample size of AIRTIVITY was informed by the results of AIRLEAF in 322 people with bronchiectasis (∼30% reduction in rate of PExs with verducatib 2.5 mg *versus* placebo) and the phase III ASPEN trial (brensocatib (DPP1 inhibitor); ∼20% reduction in rate of PExs *versus* placebo in 1721 people with bronchiectasis) [22, 29].

The design of and safety monitoring in AIRTIVITY were also informed by examining the effects of complete DPP1 absence in people with PLS, a rare genetic disease in which people have loss-of-function mutations in both alleles of the *DPP1* gene [35]. PLS is typically characterised by palmoplantar hyperkeratosis and severe periodontitis [35]; however, the absence of functional DPP1 in PLS does not lead to an increased risk of infections in most people with this condition [35]. In AIRLEAF, AEs potentially related to the mode of action (PLS-typical skin events, periodontal disease and opportunistic infection AEs) were infrequent, generally evenly distributed across treatment groups and placebo, and mostly of mild or moderate intensity [22]; findings were generally similar with brensocatib [28]. A clinical picture similar to PLS is not likely with verducatib treatment, given its reversible and incomplete DPP1 inhibition compared with the lifelong, complete DPP1 deficiency observed in people with PLS. Considering the low theoretical risk of these AEs following DPP1 inhibition at therapeutic doses, AIRTIVITY evaluates these AEs as part of detailed study-specific safety assessments, as informed by AIRLEAF. Based on the favourable safety profile of verducatib, AIRTIVITY includes people with clinically stable immunodeficiency (stable for >1 year before randomisation), including those on stable treatment with immunosuppressants (stable for >1 year before randomisation) and people with stable baseline hyperkeratosis (stable for >3 months before randomisation) or periodontitis.

### Innovations in AIRTIVITY™

Considering the heterogeneity of bronchiectasis, an innovation in AIRTIVITY is the permitted enrolment of a broader population than previous large studies in bronchiectasis. Notably, people with bronchiectasis and a primary diagnosis of CF, asthma or COPD are eligible. Recent studies have indicated that those with a primary diagnosis of asthma or COPD have a high PEx burden [36, 37]. The rationale for assessing verducatib in this broad bronchiectasis population is the common histopathological and pathophysiological features across aetiologies (*e.g.* people with CF receiving CFTRmt have been shown to have similar levels of inflammation to people with bronchiectasis caused by other aetiologies) [21, 38]. Due to the role of DPP1 in activating NSPs [24, 25], and concerns generated from previous therapeutics targeting neutrophils that have impacted host defence, including in studies of people with CF [39], recent studies of DPP1 inhibitors in bronchiectasis have excluded people with immunodeficiency [28, 29]. However, given that the studies demonstrated no increase in infectious complications with DPP1 inhibitors [22, 28, 29], the AIRTIVITY study permits the inclusion of people with stable immunodeficiency or receiving immunosuppressants, including those receiving immunoglobulin replacement therapy; these individuals have been excluded from previous similar studies of DPP1 inhibitors. The inclusion of such a broad population sets the AIRTIVITY study apart from other studies in bronchiectasis [22, 29] and more accurately reflects the real-world bronchiectasis population. This aetiology-agnostic approach expands the potential for treatment opportunities in people with all forms of bronchiectasis.

Although bronchiectasis affects both adults and children, traditional drug development has prioritised the adult population. AIRTIVITY includes adolescents and, in doing so, may reduce the current inequity in clinical research for underserved young populations, and improve the generalisability of trial findings across age groups.

As symptom burden has been shown to predict PEx risk [40, 41], independent of the presence of effective registered therapies, AIRTIVITY allows enrolment of people with one PEx in the year prior to screening if they have a high symptom burden (SGRQ Symptoms score >40), in addition to people with at least two PExs in the year before screening; this is consistent with AIRLEAF, which confirmed that these two populations had a similar benefit with verducatib treatment [22]. In contrast, the ASPEN, WILLOW (phase II study of brensocatib) and SAVE-BE (phase II trial of the DPP1 inhibitor HSK31858) trials included only adults with at least two PExs in the year prior to screening [28–30].

The 2:1 allocation ratio was selected to increase the potential for participants to receive verducatib in the study. The long study duration (52–76 weeks) will provide long-term efficacy and safety data on the effect of verducatib 2.5 mg and is longer than the duration of other similar studies of DPP1 inhibitors [22, 28, 29]. Monthly phone calls between site visits (incorporated following patient feedback) and the optimised collection of patient-reported outcomes (designed with consideration of participant burden during visits) are further strengths of the study.

As discussed previously, the design of AIRTIVTY was informed by safety data from phase II studies of DPP1 inhibitors, in which detailed periodontal and skin assessments were conducted [22, 28]. Periodontal and skin assessments are included in AIRTIVITY, adapted based on experience from the AIRLEAF study [22]. Overall, these aspects of the study design contribute to its feasibility and patient-centric focus by reducing patient burden without compromising on data quality.

Finally, the study permits the use of therapies that reflect standard-of-care treatments for bronchiectasis (*e.g.* antibiotics) and its underlying conditions or comorbidities (*e.g.* CFTRmt for CF and monoclonal antibodies for severe asthma or eosinophilic COPD), enabling participants to continue pre-existing treatments during the study, better reflecting real-world treatment.

### Conclusion

The phase III AIRTIVITY study, assessing verducatib 2.5 mg in people with bronchiectasis, started in June 2025 and is being conducted in approximately 50 countries. The first adolescent was screened in March 2026. The patient-centric study design was informed by patient feedback and the methodology and results from other studies. If efficacy, safety and tolerability are demonstrated in AIRTIVITY, these results will provide confirmatory evidence for using verducatib 2.5 mg in a large and broad population of people aged ≥12 years with bronchiectasis, regardless of underlying aetiologies, considering symptom burden as a predictor for disease deterioration.

## Data Availability

To ensure independent interpretation of clinical study results and enable authors to fulfill their role and obligations under the ICMJE criteria, Boehringer Ingelheim grants all external authors access to clinical study data pertinent to the development of the publication. In adherence with the Boehringer Ingelheim policy on transparency and publication of clinical study data, scientific and medical researchers can request access to clinical study data, typically 1 year after approval has been granted by major regulatory authorities or after termination of the development programme. Researchers should use https://vivli.org/ to request access to study data and visit www.mystudywindow.com/msw/datasharing for further information.
